# Synthetic-lattice enabled all-optical devices based on orbital angular momentum of light

**DOI:** 10.1038/ncomms16097

**Published:** 2017-07-14

**Authors:** Xi-Wang Luo, Xingxiang Zhou, Jin-Shi Xu, Chuan-Feng Li, Guang-Can Guo, Chuanwei Zhang, Zheng-Wei Zhou

**Affiliations:** 1 Key Laboratory of Quantum Information, University of Science and Technology of China, Hefei, Anhui 230026, China; 2 Department of Physics, The University of Texas at Dallas, Richardson, Texas 75080-3021, USA; 3 Synergetic Innovation Center of Quantum Information and Quantum Physics, University of Science and Technology of China, Hefei, Anhui 230026, China

## Abstract

All-optical photonic devices are crucial for many important photonic technologies and applications, ranging from optical communication to quantum information processing. Conventional design of all-optical devices is based on photon propagation and interference in real space, which may rely on large numbers of optical elements, and the requirement of precise control makes this approach challenging. Here we propose an unconventional route for engineering all-optical devices using the photon’s internal degrees of freedom, which form photonic crystals in such synthetic dimensions for photon propagation and interference. We demonstrate this design concept by showing how important optical devices such as quantum memory and optical filters can be realized using synthetic orbital angular momentum (OAM) lattices in degenerate cavities. The design route utilizing synthetic photonic lattices may significantly reduce the requirement for numerous optical elements and their fine tuning in conventional design, paving the way for realistic all-optical photonic devices with novel functionalities.

## Abstract (web summary)

Design of all-optical devices rely on large numbers of optical elements and precise control makes this approach challenging. The authors demonstrate that optical devices such as quantum memory and optical filters can be realized using synthetic orbital angular momentum lattices in a single main degenerate cavity.

The ability to coherently control the properties of photons, such as their storage and propagation, is crucial for many important technological applications in various fields, ranging from optical communications^1^,^2^,^3^, data storage^4^,^5^,^6^,^7^, to quantum information processing^8^,^9^. The devices used for such purpose may involve the interaction of photons with other physical media (for example, atoms)^7^,^9^ or contain only optical elements, that is, all-optical photonic devices^2^,^3^,^4^,^5^,^6^. In conventional all-optical devices, photonic properties are controlled through the manipulation of the photon interference in the real space^3^,^5^. Typical examples include photonic crystals, where coupled arrays of photonic circuits are implemented by fine tuning the parameters of associated optical elements. The conventional all-optical photonic devices have been extensively studied and showcase great applications, ranging from practical devices^2^,^5^,^6^ to fundamental topological photonics^10^,^11^,^12^. However, such real-space photonic devices usually demand precise control of a large number (∼100) of spatially separated optical elements (for example, resonators, waveguides and so on), which can be very complicated and resource-costing for many practical applications.

On the other hand, photons possess many internal degrees of freedom (for example, frequency, polarization, orbital angular momentum (OAM)^13^,^14^ and so on), which may form synthetic-lattice dimensions for photons (that is, synthetic photonic crystals) in addition to real space. Recently, synthetic-lattice dimensions have been explored in ultra-cold atomic gases with the direct experimental observation of quantum Hall edge states^15^,^16^,^17^. Among photon’s internal states, OAM has found great applications in quantum information^18^,^19^,^20^,^21^, optical communications^22^,^23^ and the realization of topological matter^24^,^25^,^26^,^27^ because of the large number of available distinctive OAM states, which also makes it one of the most promising candidates for synthetic photonic lattices. Compared with the real-space photonic crystals containing a large number of optical elements, the synthetic photonic lattices may significantly reduce the physical complexity of the system and thus are more resource-efficient.

In this article, we propose an unconventional route for engineering all-optical photonic devices based on photon propagation and interference in synthetic photonic lattices. We explore this design paradigm by showing how important photonic devices such as quantum memory and optical filters that are vital for quantum communication networking and optical signal processing can be implemented, using synthetic OAM lattices where the photon is stopped and stored. We consider a degenerate-cavity system containing only a single main cavity that supports multiple degenerate OAM modes, where the interference of photon in the OAM lattices can be manipulated by simply tuning an optical phase. The proposed all-optical quantum memory is more resource-efficient and experimentally simpler than the conventional real-space coupled-cavity-based memory that requires precise control of a large number of coupled cavities^28^. They have a large bandwidth and no restriction on the working frequency compared with the atomic-ensemble-based memory^29^. The proposed route will not only motivate other novel applications and devices based on the OAM lattices, but also open an avenue for engineering all-optical photonic devices utilizing other internal degrees of freedom (for example, frequency and so on) as synthetic-lattice dimensions^30^,^31^,^32^.

## Results

### Photon OAM and coupled-degenerate cavities

**Figure 1:**
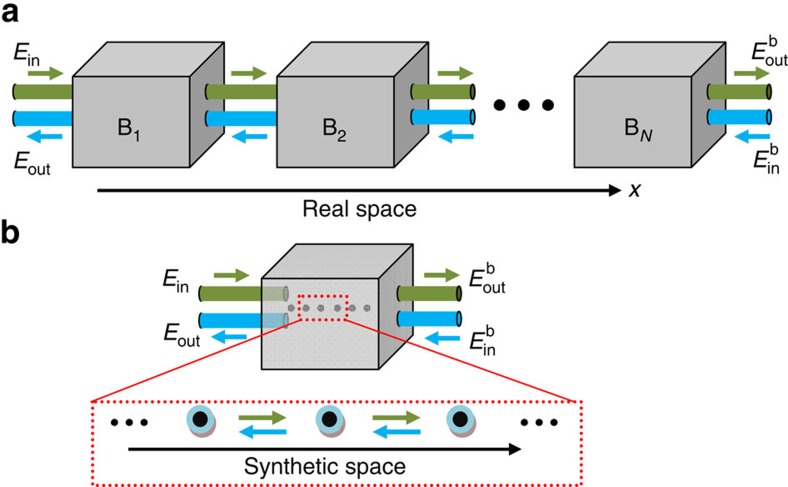
Illustration of the design principle for photonic devices. (**a**) Conventional devices such as photonic crystals based on photon interference in real space. The building blocks (boxes B_1_, B_2_, …), which consist of optical elements such as interferometers or resonators, are separated in real space and coupled by fibres or waveguides. (**b**) Unconventional devices based on photon interference in synthetic photonic lattices formed by photon’s internal degrees of freedom. The dots (lattice sites) represent different internal states. 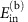 and 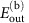 represent the optical fields in the four input/output channels.

As illustrated in Fig. 1a, the conventional real-space optical devices usually rely on large numbers of optical elements, and the requirement of precise control makes this approach challenging. In contrast, optical devices based on synthetic lattices (Fig. 1b) may significantly reduce the physical complexity of the system. Such synthetic photonic lattices can be formed by photon’s OAM, a fundamental optical degree of freedom. Solutions of the light field in an optical system with cylindrical symmetry have an angular dependence 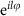, where 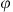 is the azimuthal angle and *l* is an integer^13^. This is a fundamental optical degree of freedom associated with the OAM of photons that has a value of *lħ* per photon^14^. In comparison with other optical degrees of freedom, OAM has a fascinating property that an infinite number of distinctive OAM states are available. These discrete OAM *l* states can be used to denote discrete lattice sites in the OAM-enabled synthetic-lattice dimensions.

**Figure 2:**
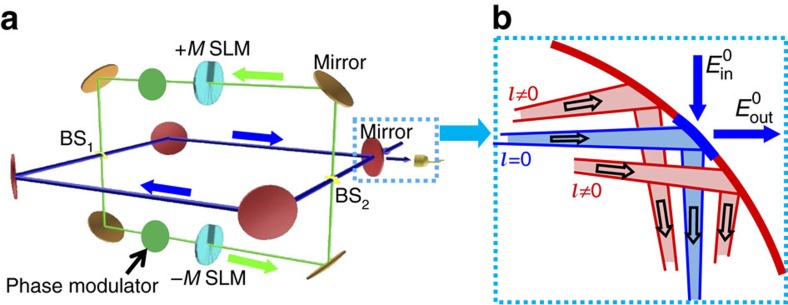
All-optical devices based on a degenerate cavity system. (**a**) The main (red) and auxiliary (brown) cavities are coupled by a pair of beam splitters (BSs), both cavities are degenerate. Two spatial light modulators (SLMs) are used to change photon’s OAM by ±*M*. (**b**) The input/output port realized as a pinhole at the centre of a mirror to couple the *l*=0 mode in and out.

The OAM-based synthetic photonic lattices can be generated and manipulated using a degenerate cavity^33^,^34^ that can support multiple OAM modes simultaneously. In experiments, such a degenerate cavity with a large number of OAM modes is easy to realize with a flexible configuration^33^. The proposed all-optical photonic devices rely on the coupling between different OAM modes in the degenerate cavity for photon interference and propagation, which can be realized using an auxiliary cavity. The optical design, shown in Fig. 2, consists of a main degenerate cavity and an auxiliary degenerate cavity coupled by two beam splitters with low reflectivity. Unlike the main cavity, the length of the auxiliary cavity is chosen for destructive interference, therefore most photons remain in the main cavity. Two spatial light modulators (SLMs) such as very low-loss vortex phase plates^35^ are inserted into the auxiliary cavity, which couple OAM mode *l* of the passing photons to its adjacent modes *l*±*M* with *M* being the step index of the SLM. Two phase modulators placed in the two arms of the auxiliary cavity generate different phases for the coupling along two arms, which can be realized using, for instance, high-speed electro-optic index modulation^13^,^36^.

**Figure 3:**
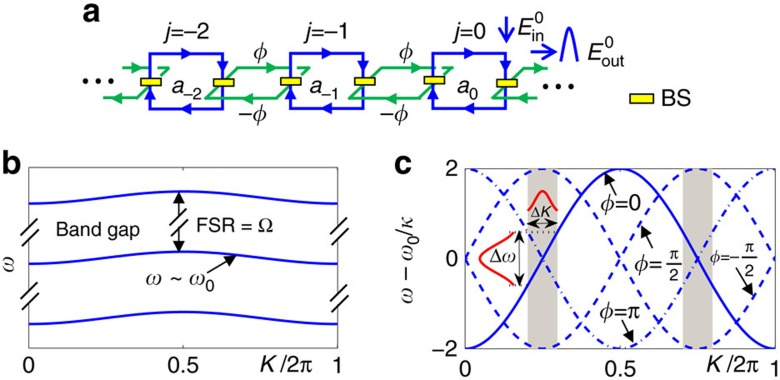
Equivalent circuit and spectrum of the proposed photonic devices. (**a**) The equivalent circuit in the OAM space, with *a*_*j*_ the field operator for OAM mode *jM* and *ϕ* the phase imbalance between the two arms of the auxiliary cavity. (**b**) Spectrum bands of the system separated by the FSR of the cavity. (**c**) Dependence of the spectrum on *ϕ*. Momentum and frequency ranges covered by the signal (Δ*K* and Δ*ω*) are marked by the red pulses.

Interestingly, the single main cavity system in Fig. 2a is conceptually equivalent to a 1D array of coupled-optical resonators^24^, which makes our scheme much simpler than previous coupled-cavity-based quantum devices that contain more than 100 cavity units each consisting of several carefully coupled and tuned cavities^28^. This mapping is illustrated in Fig. 3a, where the *j*th state with an OAM number *l*=*jM* is associated with the position index of a cavity. The beam splitters (BSs) divert a portion of the photon in the main cavity towards the SLMs and merge it back with its OAM state changed by ±*M*, which correspond to the tunnelling of a photon along the OAM lattice in Fig. 3a with the tunnelling rate determined by the reflectivity of the BSs. In the tunnelling process, the photon can also acquire a phase determined by the optical path length of two arms of the auxiliary cavity. In the weak coupling limit between auxiliary and main cavities, the Hamiltonian for the system in Fig. 3a can be written as^10^


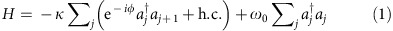


in the OAM lattices, where *a*_*j*_ is the annihilation operator of the cavity photon of OAM mode *jM*, *ϕ* is the phase imbalance between the two arms of the auxiliary cavity and *ω*_0_ is the resonant frequency of the main cavity. The tunnelling rate between OAM modes *κ*=Ω_0_*α*/[(1+*α*)2*π*], where *α*=|*r*_B_|^2^/(1+|*t*_B_|^2^), *r*_B_ and *t*_B_ are the reflectivity and transmissivity of the coupling beam splitters (Supplementary Note 1), Ω_0_=2*πc*/*L* is the free spectral range (FSR) of the cavity, *L* is the total length of the cavity optical path and *c* is the speed of light.

For the critical input and output channels of the photonic device, we introduce a low-reflectivity pinhole at the centre of the input/output mirror as shown in Fig. 2b, which can be implemented using, for instance, graded coating^37^. This is a widely used technique^22^,^23^ to differentiate *l*=0 mode from others since *l*=0 is the only mode with a high intensity at the beam centre^13^,^38^. The rotationally symmetric pinhole does not affect the OAM number of the cavity modes, and it introduces a large loss rate for the *l*=0 and low OAM modes, since they can leak out of the cavity via the pinhole and couple to outside modes. In contrast, higher OAM modes, whose field distribution has negligible overlap with the pinhole, are hardly disturbed, just like a *l*=0 mode is not affected by the finite aperture of the mirror in a cavity (without a pinhole) though its wave front is infinite in theory. With proper choice of the step index *M*, only *j*=0 mode in the cavity couples to the input/output field has a significant rate of loss.

When photons propagate in all-optical devices, the time evolution of optical modes (in Heisenberg picture) is described by^39^


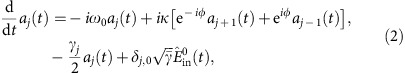


where *a*_*j*_(*t*) is the time-dependent field operator of OAM mode *jM*, *γ*_*j*_ is its loss rate and 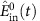 is the input field operator, which couples to the *l*=0 mode in the cavity at a rate 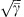 determined by the reflectivity of the input/output pinhole. The input field operator is given by 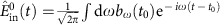, with *t*_0_→−∞ and *b*_*ω*_(*t*_0_) being the annihilation operator of the input photon with frequency *ω*. The proposed devices work for both quantum single-photon and classical coherent state input pulses (Supplementary Note 2), since the dynamics of our system is characterized by the linear equation of photon operators (equation (2)).

The system is periodic (with translational symmetry) along the OAM lattice, thus can be analysed using the Bloch theory. In particular, the eigenstates of the system are given by the Bloch functions, which are obtained by Fourier transformation of the local OAM modes (Wannier modes). We can rewrite the Hamiltonian as


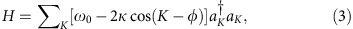


where 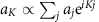 is the field operator of the corresponding Bloch function, and *K* is the Bloch wave number. The Bloch wave number is a good quantum number related to the translational symmetry, which characterizes the phase difference between neighbour OAM states in the Bloch functions (Supplementary Note 1). The dispersion spectrum is


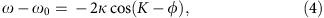


where *ω* is the system’s eigenfrequency. Clearly the dispersion relation (Fig. 3b,c) and the propagating group velocity 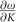 in the OAM space can be manipulated by simply tuning the phase imbalance *ϕ* in experiments. Such tunability make it possible to realize important optical devices such as quantum memory and optical filter.

### Quantum memory in synthetic OAM lattices

Quantum memory is a key element in many quantum information protocols^7^,^8^. Since information is encoded in photons in a quantum communication network, any non-optical element, such as atomic ensemble^29^, requires transferring of information from and back to photons, which complicates the operation of the quantum memory and lowers its efficiency. Furthermore, only a very limited number of elements are suitable for atomic-ensemble-based quantum memory, and the frequency range is restricted to available atomic transitions^29^. An all-optical quantum memory eliminates the need to transfer information between different physical media, and can in principle lead to simplified operation and improved efficiency. However, existing schemes for all-optical quantum memory based on coupled-optical resonators^4^,^28^ or modulation of index of refraction^5^,^40^ have their own difficulties for fabricating large numbers of identical optical cavities or homogeneously tuning the index of optical materials.

An all-optical quantum memory based on slowing/stopping light through photon interference in the OAM lattices can overcome those difficulties of existing schemes and offer compelling advantages. The photon propagation is now slowed down in the OAM-enabled synthetic lattices by tuning the phase *ϕ*, which is much simpler and more reliable compared with the simultaneous and precise tuning of hundreds of cavities for quantum memory based on coupled resonators in real dimension^28^. The major operation procedure for the quantum memory consists of three steps by controlling the phase imbalance *ϕ*: (i) writing the input signal into the memory by coupling to the *l*=0 mode in the cavity through the input pinhole; (ii) letting the signal in the cavity propagate to certain high *l*≠0 modes, and storing it there for a desired storage time; (iii) making the signal propagate back to the *l*=0 mode for read-out by coupling to the output through the same pinhole for write-in.

**Figure 4:**
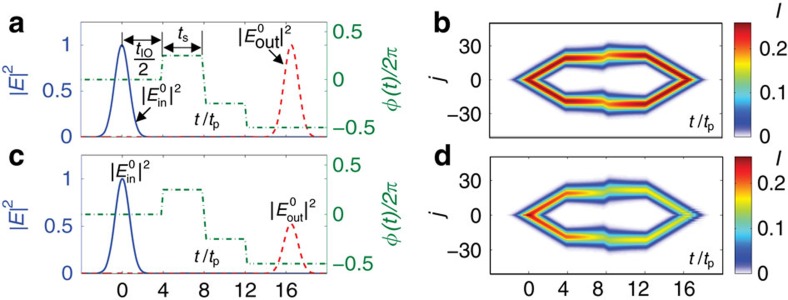
Time evolution of the optical signal in the quantum memory. (**a**) Control sequence for the phase imbalance in the auxiliary cavity and calculated output power normalized to the maximum intensity of the input pulse, assuming a Gaussian profile for the input pulse and no loss for all cavity modes except that due to the coupling to the input signal. 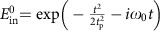 with *t*_p_=2.5*κ*^−1^ and *γ*_*j*_=*δ*_*j*,0_4*κ*. (**b**) Evolution of the optical signal’s distribution in the OAM lattices under the same assumption as in **a**. *I* is the field intensity. (**c**,**d**) The same as in **a** and **b**, except that losses of the cavity modes are taken into account by assuming *γ*_*j*_=*δ*_*j*,0_4*κ*+0.2*κ*e^−|*j*|^+0.01*κ* (Supplementary Notes 2 and 3).

For a proof-of-principle illustration of our OAM-based quantum memory, we first ignore the loss of all *l*≠0 modes and assume 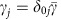. As shown in Fig. 4a,b, if we design the system such that 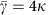, the incoming signal pulse is absorbed into the cavity with an efficiency of 100% (Supplementary Note 2). To store pulses significantly shorter than the write-in time *t*_IO_, the usable memory bandwidth 2*κ* (ref. 41) should satisfy the condition 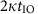
≳12*π* (ref. 42). Once in the cavity, all frequency components of the signal pulse start to propagate to *l*≠0 modes. For a long storage time, the signal may propagate to high OAM numbers. Although there is no theoretical upper limit for the OAM number, in reality, it is limited by the practical factors such as the aperture size of the optical elements in the cavity.

To limit the OAM number, we slow down the propagation of the signal pulse in the OAM lattices by tuning the phase imbalance *ϕ*. As illustrated in Fig. 4a,b, *ϕ* is set to 0 in the write-in process. When the signal pulse enters the cavity completely after a write-in time *t*_IO_, its peak travels (in the OAM lattices) approximately at a group velocity 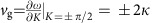. We then change the phase to *ϕ*=*π*/2 adiabatically compared with the bandgap of the system approximately given by the FSR (Fig. 3b). The modulation of *ϕ* preserves the system’s translational symmetry in the OAM lattices, and thus the Bloch wave number of the signal is conserved. At *ϕ*=*π*/2, *v*_g_ becomes 0 (Fig. 3c), and the pulse stops propagating in the OAM lattices as shown in Fig. 4b.

Meanwhile, the pulse starts to expand in the OAM lattices due to the dispersion of the spectrum, which causes distortion in the temporal profile of the signal. To correct this distortion and restore the signal to its original shape for read-out, we tune *ϕ* to −*π*/2 and keep its value at −*π*/2 for the same amount of time *t*_S_ for which *ϕ* was set to *π*/2. Finally, we tune *ϕ* to −*π*. As shown in Fig. 4a,b, after another period of time equal to the write-in time *t*_IO_, the above phase echo procedure not only returns all frequency components of the signal to the *l*=0 mode, but also corrects any distortion accumulated in the first half of the process. The pulse can be read-out with an efficiency of 100% under the condition 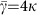 and the total storage time 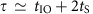. To ensure full emission, it is required that 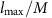
≳
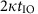
≳12*π*, with *l*_max_ the maximum OAM state that the cavity can support.

**Figure 5:**
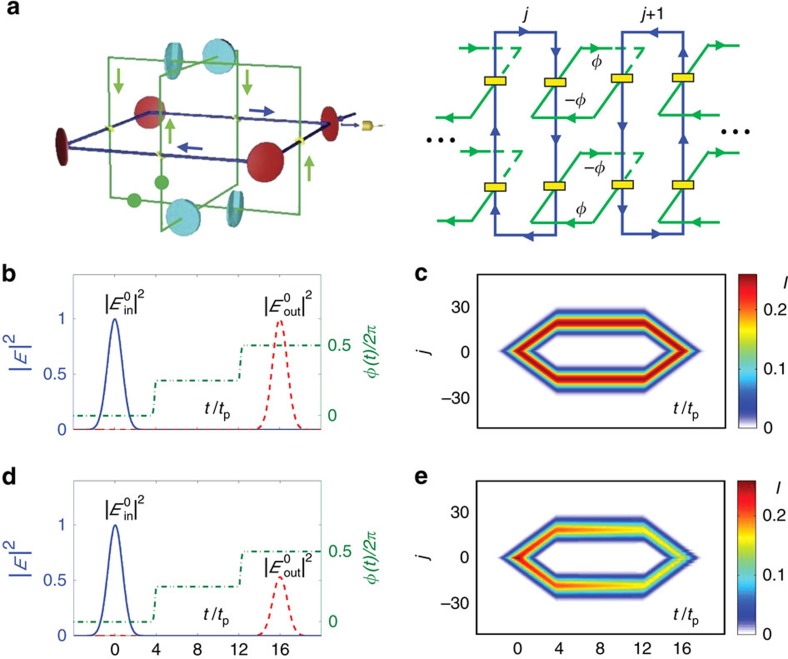
Modified quantum memory allowing on-demand recall. (**a**) The system with two auxiliary cavities and its equivalent optical circuit in the OAM space. The phase imbalances of the two auxiliary cavities are opposite to each other. (**b**–**e**) Same as Fig. 4a–d except with two auxiliary cavities and slightly different control sequence for the phase imbalance *ϕ*.

Although the storage time of our OAM-based quantum memory is controllable, it is preset. We can freeze the photon signal in the OAM lattices and enable its on-demand recall by slightly modifying our design from the device in Fig. 2. The corresponding circuits are shown in Fig. 5a, which use two auxiliary cavities with the same coupling strength *κ*/2 and opposite phase imbalances ±*ϕ*. Because of the interference between the two auxiliary cavities, the dispersion relation of the system becomes


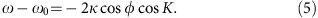


The group velocities of the pulse peaks at *K*=±*π*/2 become *v*_g_=±2*κ* cos *ϕ*. Once the input signal is absorbed into the cavity, we can stop the pulse’s propagation and dispersion in the OAM lattices completely by adiabatically changing *ϕ* from 0 to *π*/2, which compresses the bandwidth to 0 because transitions between OAM modes via the two auxiliary cavities cancel each other. As shown in Fig. 5b,c, the optical signal and its distribution in the OAM lattice can then be kept for an arbitrary and indefinite amount of time until it needs to be read-out by changing *ϕ* from *π*/2 to *π*. This allows the on-demand recall of the photon signal and random access to the quantum information that it carries. The storage fidelity of a single-photon pulse, defined as the wave-packet overlap between input and output conditional on the re-emission of a photon^29^, can be as high as 1. Thus, it is possible to realize perfect write-in, storage and on-demand read-out of an optical signal using only a limited number of OAM states sufficient for the signal pulse to be absorbed into the cavity.

In reality, all OAM modes are lossy due to factors such as intrinsic loss of the optical elements and leakage of *l*≠0 modes via the input/output pinhole. It is demonstrated that (Supplementary Notes 2 and 3) our OAM-based quantum memory still functions as expected without wave-packet distortion in the presence of imperfections, although the efficiency is reduced as shown in Figs 4c,d and 5d,e. The single-photon storage fidelity remains as high as 99.95% in Fig. 5d.

For the estimation of experimental parameters, we assume that the cavity is realized using four curved mirrors each with a focal length *F* on the order of centimetres, a typical value for discrete optical elements. Since the separation between the mirrors is 2*F*, the total length is about tens of centimetres for the optical path, which gives a FSR (Ω_0_) of 2*π* × 0.5 GHz–2*π* × 1.0 GHz. By choosing a proper reflectivity 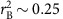 for the beam splitters, we estimate that the total bandwidth 4*κ* is about 2*π* × 50 MHz–2*π* × 100 MHz. Therefore, the quantum memory can store short pulses with a temporal duration of tens of nanoseconds. The bandwidth can be further improved by using a smaller focal length *F*. The required modulation time for the phase imbalance *ϕ* is also on the order of tens of nanoseconds, consistent with the modulation speed of current electro-optic devices^13^,^36^. With a photon loss rate of the order of MHz, the storage time is about 1 μs (Supplementary Note 3). As a comparison, the storage time of ideally identical coupled-micro-resonator-based memory is limited below 0.1 μs due to the large photon losses of the micro-resonators (about tens of MHz)^28^.

### OAM-enabled optical filter

We can build upon our ideas to envision further interesting and valuable applications. One such example is high skirt-slope optical filters, which are crucial in many fields such as quantum information^43^,^44^,^45^, high-density wavelength-division-multiplexing networking and optical signal processing^46^,^47^,^48^. For good selectivity, the filter function should ideally have a narrow bandwidth and a steep skirt slope at the edge of the stopband. It is then critical to improve the shape factor, which is often evaluated by the ratio of the stopband width at −25 and −3 dB (refs 47, 48). Conventionally, this is usually achieved by coupling many carefully designed cavities to obtain a high-order filter^48^. Because of inevitable errors in fabrication and tuning, the number of cavities that can be reliably coupled in practice is quite limited. Consequently, it is very challenging to realize high-shape factors in an optical filter based on many coupled cavities.

**Figure 6:**
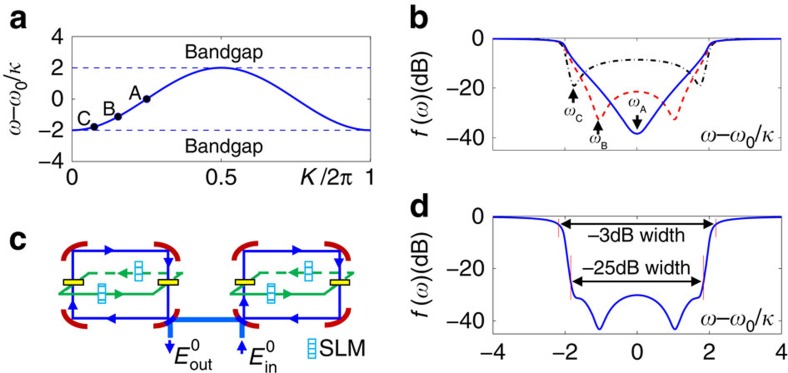
Design of a stopband optical filter based on photon’s OAM. (**a**) Spectrum band of the device in Fig. 2 with three marked frequencies *ω*_A_=*ω*_0_, *ω*_B_≃*ω*_0_−1.1*κ* and *ω*_C_≃*ω*_0_−1.8*κ*. The corresponding group velocities are *v*_g_(*ω*_A_)=2*κ*, *v*_g_(*ω*_B_)=1.65*κ* and *v*_g_(*ω*_C_)=0.9*κ*. (**b**) The filter function 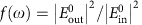 of the device in Fig. 2 when its maximum absorption frequency is designed to be *ω*_A_, *ω*_B_ and *ω*_C_, respectively. A maximum absorption frequency closer to the band edge results in a steeper skirt slope but poorer in-band rejection ratio. (**c**) A two-cavity design with their maximum absorption frequencies chosen to be *ω*_C_ and *ω*_B_, respectively. (**d**) The filter function for the design in **c**. (**b**,**d**) 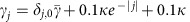.

It is possible to achieve an optical filter with very high-shape factors using the band spectrum equation (4) generated by the photon interference in the OAM lattices in Fig. 6. The filter characteristics can be obtained by analysing the wave propagation in the coupled many-cavity system in Fig. 3a, which is a conceptual equivalent to our OAM-based device. However, a much more intuitive understanding based on the system spectrum is possible, which can greatly facilitate the design of the filter to obtain desired properties. In the device in Fig. 2, when an *l*=0 signal is fed to the input/output port, all frequency components in the bandgaps cannot enter the cavity and are reflected. The cavity absorbs in-band frequency components with an efficiency dependent on the coupling rate 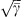 and the group velocity *v*_g_(*ω*) of the *l*=0 cavity mode in the OAM lattices. The maximum absorption occurs at the frequency *ω*_m_ determined by 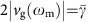 (Supplementary Note 2). If we choose the reflectivities of the coupling beam splitter and input/output pinhole appropriately such that *ω*_m_ is very close to the cavity’s band edge *ω*_e_=*ω*_0_±2*κ*, the cavity changes from being totally reflective to being strongly absorptive to the incident light over a narrow frequency range |*ω*_m_−*ω*_e_|. This results in a desired steep skirt slope as shown in Fig. 6b. Unfortunately, because of the frequency dependence of the group velocity, for such a choice of *ω*_m_ the absorption is poor at the centre of the stopband, leading to an insufficient in-band rejection ratio, which manifests as the hump at the bottom of the filter function in Fig. 6b.

To overcome this difficulty, we use the two-cavity design in Fig. 6c. While the maximum absorption frequency of the first cavity is still chosen to be close to the band edge, that of the second cavity is chosen closer to the centre of the stopband to suppress the hump in Fig. 6b. Such a design results in a narrow and deep stopband with sharp edges, which is ideal for optical filters.

Since the input and output fields are both in the *l*=0 mode, the filter function for our filter is calculated by ref. 24


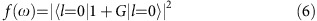


with


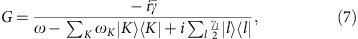


where |*l*〉 is an OAM state, |*K*〉 is a Bloch state in the OAM lattices with frequency *ω*_*K*_=*ω*_0_−2*κ* cos *K*. As plotted in Fig. 6d, a shape factor of 0.85 can be realized with just moderate SLM and cavity efficiencies (Supplementary Note 4), which is noticeably higher than current technologies that are limited by the number of high-Q cavities that can be reliably coupled in practice^48^,^49^,^50^.

## Discussion

Because of the unlimited range of the OAM space, the photon OAM is recognized as a unique asset for many applications in quantum information and optical communications. Generally, a large number of optical elements need be arranged and precisely controlled in real space to utilize many OAM states, which set up a major limitation for many applications using traditional photonic devices illustrated in Fig. 1a. For instance, in the study of quantum random walk in the OAM space, each walk step requires a cascaded optical stage. The number of optical elements (*q*-plates or SLMs) increases linearly with the number of walk steps^25^,^26^,^27^ (although already an improvement from other schemes), resulting in complex and very challenging optical set-up to reach many OAM states. In contrast, our synthetic photonic OAM lattice does not suffer the limitation induced by the large number of optical elements because the degenerate-cavity system requires only a few optical elements without the need of many cascaded optical stages, and high OAM states can be generated by passing photons through one SLM many times. As long as high-performance optical elements are used to construct the designed system with a high quality, one would be able to use many OAM states (for example, hundreds). Moreover, a higher OAM state has a larger beam size, thus the upper limit of usable OAM state is determined by the aperture size of the optical elements in the cavity, which can be extremely high. Other imperfections such as photon losses will also limit the propagation distance of photons in the synthetic OAM space, and degrade the performance of the optical devices, as discussed in the following.

Intrinsic loss due to the finite finesse of the cavity can be very low as long as high quality cavities are used^51^. Photon losses can also be introduced by phase modulators due to absorption by their optical media and SLMs because of their limited resolution and fabrication error. Such losses can be made very low^35^,^52^,^53^,^54^ (Supplementary Note 3), and further reduced by the fact that the auxiliary cavity is designed using destructive interference with very few photons in it. The low-reflectivity pinhole manifests as an photon loss that decreases rapidly with the OAM number (Supplementary Note 3). For the quantum memory, the storage time (for a fixed storage efficiency) decreases rapidly with the increase of photon losses. Photon losses due to the phase modulators and SLMs are the limiting factors for the storage time, since their effect is persisting, even during the storage phase when the signal is frozen at large OAM states. We find that for the optical filter, the shape factor and stop bandwidth are less sensitive to imperfections of these optical elements, as confirmed by our numerical simulation.

Even with the limitations posed by these practical considerations, the OAM-based quantum memory has a few attractive characteristics and noteworthy advantages that are not available in existing schemes. Not only is the system very simple with just a single main cavity and thus completely realizable with conventional optical technology, but also the operating wavelength can be chosen at will, a significant edge in situations where no atomic systems with the desired transition frequency are available. It is also not limited by the technical challenge to fabricate and tune many identical optical cavities^28^. The bandwidth of the quantum memory and its storage time are limited by the size of the cavity, and loss of the SLMs and phase modulators, instead of the delay-bandwidth product of the system or other intrinsic factors. Finally, polarization-independent optical elements can be used so that information encoded in both temporal wave-packet and polarization can be recovered with fidelity close to 1.

In conclusion, we propose a conceptually unconventional route for engineering all-optical photonic devices based on photon propagation and interference in synthetic lattices. We demonstrate this design principle by showing that two powerful devices, quantum memory and an optical filter, can be realized utilizing photon OAM-based synthetic lattices. The proposed route may inspire new and simple designs for many other photonic devices (for example, multi-channel optical router and so on), and open an unconventional avenue for photonic technology and applications.

### Data availability

The data that support the findings of this study are available from the corresponding author upon reasonable request.

## Additional information

**How to cite this article:** Luo, X.-W. *et al*. Synthetic-lattice enabled all-optical devices based on orbital angular momentum of light. *Nat. Commun.* **8,** 16097 doi: 10.1038/ncomms16097 (2017).

**Publisher’s note**: Springer Nature remains neutral with regard to jurisdictional claims in published maps and institutional affiliations.

## Supplementary Material

Supplementary Information
