# Supplementary material for: Synthetic-lattice enabled all-optical devices based on orbital angular momentum of light
Source: Nat Commun. 2017 Jul 14;8:16097. doi: 10.1038/ncomms16097 (PMC5519981; doi:10.1038/ncomms16097)
Supplement: Supplementary Information [file ncomms16097-s1.pdf]

File name: Supplementary Information
Description: Supplementary Figures, Supplementary Notes and Supplementary References.

Supplementary Note 1: The tight-binding Hamiltonian

As explained in the main text, our degenerate-cavity system is conceptually equivalent to a 1D array of coupled cavities shown in Supplementary Figure 1. We consider the eigenmode field E which satisfies the Maxwell equation

$$\nabla \times (\nabla \times E) = \epsilon(\mathbf{r})\frac{\omega^2}{c^2}E, \tag{1}$$

where $\epsilon(\mathbf{r})$ is the dielectric constant of the system and ω is the eigenfrequency. For weak coupling between cavities, E can be expanded in local Wannier modes [1–3],

$$E = \sum_j \psi_j W_j(\mathbf{r}), \tag{2}$$

where j is the lattice site index. W_j, the Wannier mode localized at site j, satisfies the Maxwell equation

$$\nabla \times (\nabla \times W_j) = \epsilon_0(\mathbf{r} - \mathbf{R}_j)\frac{\omega_0^2}{c^2}W_j \tag{3}$$

with $\epsilon_0(\mathbf{r} - \mathbf{R}_j)$ the dielectric constant at site j, ω_0 the single-site resonance frequency, and $\mathbf{R}_j$ the lattice vector of site j.

From equations (1), (2), and (3), we have

$$-\sum_{j'} \kappa_{j;j'}\psi_{j'} = (\omega - \omega_0)\psi_j, \tag{4}$$

where

$$\kappa^*_{j';j} \equiv \kappa_{j;j'} = \int d\mathbf{r}\frac{\omega_0}{2}[\epsilon(\mathbf{r}) - \epsilon_0(\mathbf{r} - \mathbf{R}_{j'})]W^*_j W_{j'}. \tag{5}$$

We have used the weak coupling condition $\kappa_{j;j'}/\omega_0 \ll 1$, and kept only leading-order terms in $\kappa_{j;j'}/\omega_0$. The on-site energy shift and non-adjacent tunneling are usually negligibly small compared to the tunneling between adjacent cavities, and we will only keep $\kappa_{j;j\pm1}$. The integration in equation (5) is limited to the region where Wannier functions have appreciable overlap, that is on the beam splitters. The system is periodic (with translational symmetry) and the coupling rate is independent of the site index, thus we have $\kappa_{j;j+1} = \kappa e^{i\phi}$. The tunneling phase ϕ comes from the phases of the Wannier functions on the beam splitters, which are determined by the phase imbalance between the two arms in the auxiliary cavity [4]. Equation (4) then leads to the following tight-binding Hamiltonian

$$H = -\kappa \sum_j (e^{-i\phi}a^\dagger_j a_{j+1} + h.c.) + \omega_0 \sum_j a^\dagger_j a_j. \tag{6}$$

By a Fourier transformation, the Hamiltonian can be written as

$$H = \sum_K [\omega_0 - 2\kappa\cos(K - \phi)]a^\dagger_K a_K, \tag{7}$$

with $a_K \propto \sum_j a_j e^{iKj}$. Thus we obtain the eigenfrequency

$$\omega = \omega_0 - 2\kappa\cos(K - \phi) \tag{8}$$

and corresponding eigenmode

$$E \propto \sum_j W_j(\mathbf{r})e^{iKj}. \tag{9}$$

The eigenmode is nothing but the Bloch function with the Bloch wave number K. The Bloch wave number (also known as Bloch momentum) is a good quantum number related to the translational symmetry. It characterizes the phase difference between neighboring OAM states (Wannier modes).

We now derive the dependence of the tunneling coefficient on the BS reflectivity, which can be accomplished by using the transfer matrix analysis [4, 5]. We introduce the photon field amplitudes c_j and c^{b}_j at each lattice site j (see Supplementary Figure 1). According to the transfer matrix formalism and Bloch theorem [4, 5], we have

$$\begin{bmatrix} c_{j+1} \\ c^{\mathrm{b}}_{j+1} \end{bmatrix} = M \begin{bmatrix} c_j \\ c^{\mathrm{b}}_j \end{bmatrix} = \begin{bmatrix} c_j \\ c^{\mathrm{b}}_j \end{bmatrix} e^{-iK}, \tag{10}$$

with the Bloch momentum K and the transfer matrix

$$M = \begin{bmatrix} e^{-ikS_{\mathrm{c}}} & 0 \\ 0 & e^{ikS_{\mathrm{c}}} \end{bmatrix} M_{\mathrm{B}} e^{-i\phi} \begin{bmatrix} e^{-ikS_{\mathrm{a}}} & 0 \\ 0 & e^{ikS_{\mathrm{a}}} \end{bmatrix} M_{\mathrm{B}}. \tag{11}$$

M_{B} is the transfer matrix of the BS

$$M_{\mathrm{B}} = \begin{bmatrix} \frac{1}{-i|r_{\mathrm{B}}|} & \frac{|t_{\mathrm{B}}|}{i|r_{\mathrm{B}}|} \\ \frac{|t_{\mathrm{B}}|}{-i|r_{\mathrm{B}}|} & \frac{1}{i|r_{\mathrm{B}}|} \end{bmatrix}, \tag{12}$$

with r_{B} and t_{B} the reflection and transmission coefficients. $2S_{\mathrm{c}}$ and $2S_{\mathrm{a}}$ are the total optical path length of the main cavity and the auxiliary cavity, which are chosen for constructive and destructive interference at frequency ω_0, respectively. $k = \omega/c$ with c the speed of light. By solving equation (10), we obtain the dispersion relation

$$\omega - \omega_0 = -\frac{\Omega_0\alpha}{\pi[1 + \frac{\Omega_0}{\Omega_{\mathrm{a}}}\alpha + O(\alpha^2)]}\cos(K - \phi). \tag{13}$$

with $\alpha = |r_{\mathrm{B}}|^2/\left(1 + |t_{\mathrm{B}}|^2\right) \ll 1$, $\Omega_0 = \pi c/S_{\mathrm{c}}$ and $\Omega_{\mathrm{a}} = \pi c/S_{\mathrm{a}}$. Typically, we have $\Omega_0 \simeq \Omega_{\mathrm{a}}$, thus the tunneling coefficient is $\kappa = \frac{\Omega_0\alpha}{2\pi(1+\alpha)} + O(\alpha^3)$.

Supplementary Note 2: Efficiency for absorption and emission of the photon signal

The photon signal to be stored has a finite bandwidth around a peak frequency ω_0, which matches the resonant frequency of the main cavity. When the signal enters the

cavity, the occupied central Bloch momentum is determined by solving

$$2\kappa\cos(K-\phi)=0. \tag{14}$$

$\phi=0$ during the write-in process, yielding $K_\alpha=\pi/2$ and $K_\beta=-\pi/2$. Consequently, two pulses with Bloch momenta K_α and K_β start to propagate in the OAM lattice with opposite group velocities

$$v_{\rm g}=\frac{\partial\omega}{\partial K}|_{K=\pm\frac{\pi}{2}}=\pm 2\kappa. \tag{15}$$

The read-out process, during which ϕ is set to π, is the time reversal to write-in. The velocities of the signal in the OAM lattice are reversed and the signal propagates back to the $l=0$ mode as shown in Fig. 4a in the main text.

We analyze the read-out efficiency assuming that the only loss of the cavity modes is due to coupling to the input and output fields, i.e. $\gamma_j=\delta_{j,0}\bar{\gamma}$ with the coupling rate $\bar{\gamma}=\frac{1-\sqrt{R_{\rm h}}}{\pi\sqrt[4]{R_{\rm h}}}\Omega_0$ [6] determined by the intensity reflectivity $R_{\rm h}$ of the input/output pinhole and the free spectral range (FSR) Ω_0 of the cavity. Since only $l=0$ mode is coupled with the input and output fields, the cavity field equation (in Heisenberg picture) is

$$\begin{aligned}\frac{d}{dt}a_j(t) &= -i\omega_0+i\kappa[e^{i\phi}a_{j+1}(t)+e^{-i\phi}a_{j-1}(t)]\\ &\quad -\frac{\gamma_j}{2}a_j(t)+\delta_{j,0}\sqrt{\bar{\gamma}}\hat{E}^0_{\rm in}(t),\end{aligned} \tag{16}$$

with $\hat{E}^0_{\rm in}(t)$ the field operator of the input/output channel. In the ideal case $\gamma_j=\delta_{j,0}\bar{\gamma}$, the cavity only couples with the input/output channel, however, imperfections would induce unwanted couplings between the cavity and the dissipative channels (i.e., the vacuum state outside the cavity). In general we can rewrite equation (16) as

$$\begin{aligned}\frac{d}{dt}a_j(t) &= -i\omega_0+i\kappa[e^{i\phi}a_{j+1}(t)+e^{-i\phi}a_{j-1}(t)]\\ &\quad -\frac{\gamma_j}{2}a_j(t)+\delta_{j,0}\sqrt{\bar{\gamma}}\hat{E}^0_{\rm in}(t)\\ &\quad +\sqrt{\gamma_j-\delta_{j,0}\bar{\gamma}}\hat{E}^j_{\rm d}(t).\end{aligned} \tag{17}$$

with $\hat{E}^j_{\rm d}(t)$ the field operators of the dissipative channels. The coupling with the dissipative channels vanishes in the ideal case $\gamma_j=\delta_{j,0}\bar{\gamma}$, and equation (17) would be reduced to equation (16).

For a quantum single-photon pulse, the state of the system (in Schrödinger picture) is $|\Psi(t)\rangle=\sum_j\alpha_j(t)|1_j\rangle+\int d\omega\widetilde{E}^0(\omega,t)|1_\omega\rangle+\alpha_{\rm decay}(t)|1_{\rm decay}\rangle$, with $|1_j\rangle=a^\dagger_j|0\rangle$ being the state of a single photon occupying the j-th mode, $|1_\omega\rangle=b^\dagger_\omega|0\rangle$ being the single photon state in the input/output channel with frequency ω, and $|1_{\rm decay}\rangle$ being the single photon state in the dissipative channels. Initially $\alpha_{\rm decay}(t_0)=0$ and $\alpha_j(t_0)=0$ $(t_0\to-\infty)$ for all j. The coefficients α_j obey the same dynamics as equation (17) with a_j replaced by $\alpha_j(t)$, $\hat{E}^j_{\rm in}(t)$ replaced by the temporal input-pulse $E^0_{\rm in}(t)=\frac{1}{\sqrt{2\pi}}\int d\omega\widetilde{E}^0(\omega,t_0)e^{-i\omega(t-t_0)}$, and $\hat{E}^j_{\rm d}(t)$ replaced by zeros. The temporal output-pulse, $E^0_{\rm out}(t)=\frac{-1}{\sqrt{2\pi}}\int d\omega\widetilde{E}^0(\omega,t_1)e^{-i\omega(t-t_1)}$ with $t_1\to+\infty$, can be obtained as $E^0_{\rm out}(t)=\sqrt{\bar{\gamma}}\alpha_0(t)-E^0_{\rm in}(t)$ [7]. $\alpha_{\rm decay}(t)$ does not affect the dynamical equation of the input/output signal, it characterizes the probability of finding the single photon in the dissipative channels, with its final value given by

$$|\alpha_{\rm decay}(+\infty)|^2=\int_{-\infty}^{+\infty}dt\left(|E^0_{\rm in}(t)|^2-|E^0_{\rm out}(t)|^2\right). \tag{18}$$

For the quantum memory of single photon pulses, the efficiency and fidelity are two important criteria for assessing its performance. The efficiency is the probability to re-emit a photon that has been stored,

$$\text{Efficiency}=\frac{\int dt_{\rm o}|E^0_{\rm out}(t_{\rm o})|^2}{\int dt_{\rm i}|E^0_{\rm in}(t_{\rm i})|^2}. \tag{19}$$

The integral of $t_{\rm i}$ ($t_{\rm o}$) is taken over the input (output) duration only. The fidelity is defined as the wave-packet overlap between input and output pulse conditional on the re-emission of a photon [8],

$$\text{Fidelity}=\frac{|\int dt_{\rm i}E^0_{\rm in}(t_{\rm i})E^0_{\rm out}(t_{\rm i}+\tau)|^2}{\int dt_{\rm i}|E^0_{\rm in}(t_{\rm i})|^2\cdot\int dt_{\rm o}|E^0_{\rm out}(t_{\rm o})|^2}, \tag{20}$$

with τ the storage time. We find that the fidelity of our quantum memory is close to 1. The fidelity is related to the distortion of the signal, it is independent of the storage time (unlike the efficiency which decreases with storage time due to photon loss), since the shape of the pulse is unchanged during storage (see Fig. 5 in the main text).

For a classical coherent state pulse, the state of the system (in Schrödinger picture) now becomes $|\Psi(t)\rangle=\bigotimes_j|\alpha_j(t)\rangle\otimes|\mathcal{E}(t)\rangle$, where $|\alpha_j(t)\rangle$ is the coherent state of the j-th mode in the cavity satisfying $a|\alpha_j(t)\rangle=\alpha_j(t)|\alpha_j(t)\rangle$, and $|\mathcal{E}(t)\rangle$ is the classical coherent photon state outside the cavity, satisfying $b_\omega|\mathcal{E}(t)\rangle=\widetilde{E}^0(\omega,t)|\mathcal{E}(t)\rangle$. The coefficients $\alpha_j(t)$ and $\widetilde{E}^0(\omega,t)$ obey the same dynamics and same input/output relation as that for the single photon pulse because the dynamics of our system is characterized by the linear equation of photon operators [see equations (16) (17)].

The condition $E^0_{\rm in}(t)=0$ during the read-out leads to $E_{\rm out}(t)=\sqrt{\bar{\gamma}}\alpha_0(t)$. In a short time interval $[t,t+dt]$ during the read-out, the number of photons emitted from the $l=0$ cavity mode into the output field is $\Delta N_1=|E^0_{\rm out}(t)|^2dt=\bar{\gamma}|\alpha_0(t)|^2dt$. Meanwhile, the number of photons in the cavity that propagate from $l\neq 0$ modes back to the $l=0$ mode is $\Delta N_2=2|v_{\rm g}||\alpha_0(t)|^2dt$. When

$$\bar{\gamma}=2|v_{\rm g}|, \tag{21}$$

we have $\Delta N_1 = \Delta N_2$, implying that the signal is read out with 100% efficiency [9] in the ideal case. Similar analysis applies to the write-in process. This conclusion is confirmed by the simulations in Fig. 4a in the main text which are obtained by numerically solving equation (16).

Supplementary Note 3: Photon loss

Imperfections such as photon loss will degrade the performance of the quantum memory and limit its storage time. There are 4 major sources of photon loss in our system due to:

- Finite finesse of the cavity;
- Absorption by the phase modulator;
- Limited efficiency of the SLMs;
- Leakage through the input/output pinhole.

In the following, we analyze each loss mechanism and evaluate the overall effect of the photon loss on the performance of the quantum memory.

Intrinsic loss due to the finite finesse of the cavity can be very low as long as high quality cavities are used. Experimentally, finesse as high as $\mathcal{F} = 10^5$ has been demonstrated in ring-type optical cavities [10]. For a free spectral range $\Omega_0 \approx 1$ GHz, the corresponding intrinsic cavity loss $\gamma^{\mathcal{F}} \equiv \frac{\Omega_0}{\mathcal{F}}$ is on the order of 10 kHz. Much smaller than that from other sources, such loss is not expected to be the limiting factor for the storage time of the quantum memory.

The phase modulators introduce photon loss due to absorption by their optical media. With very low-loss material [11], such loss can be made lower than 10^{-3} for a medium thickness sufficient to generate a phase shift of π required in our control protocol. The SLMs used to increase/decrease the OAM number introduce photon loss too because of their limited resolution and fabrication error. Such loss can be made very low as the efficiency of the SLMs can be very close to 100% with appropriate design and experimental techniques [12–14]. The effect of the phase modulator and SLM loss is further reduced by the fact that the auxiliary cavity is designed with destructive interference with very little photon signal. The effective photon loss for the main cavity field caused by the phase modulators and SLMs can be characterized by an overall decay rate [6]

$$\gamma^{\mathrm{A}} \simeq \frac{|r_{\mathrm{B}}|^2}{2} \cdot \Omega_0 \frac{2(1-\sqrt{\eta^{\mathrm{A}}})}{\pi} = 4 \times (1-\sqrt{\eta^{\mathrm{A}}})\kappa, \quad (22)$$

where r_{B} is the reflectivity of the coupling beam splitter and η^{A} is a parameter close to 1 determined by the phase modulator and SLM loss.

The rotationally symmetric input/output pinhole does not affect the OAM number of the cavity modes, though it may slightly modify the radial field distribution of low OAM modes. It can introduce photon loss in two ways. First, a small portion of the $l = 0$ input field falls outside of the pinhole and cannot enter the cavity regardless of the size of the pinhole since in theory the radius of the field distribution for a Gaussian pulse is infinite. Second, a small portion of the $l \neq 0$ modes in the cavity can leak out of the cavity via the pinhole. The leakage is more serious for low l modes, and less severe for higher l modes whose peak field distribution is farther away from the center. These loss channels cannot be overcome by simply increasing or decreasing the radius of the pinhole. Doing so will alleviate one mechanism but make the other worse. It is possible to improve both by using SLMs with a larger step index $M > 1$ and a pinhole large enough to couple in most of the Gaussian input field. The consequence of doing so is that the SLMs change the OAM number of the passing light beam by $\Delta l = M > 1$, and the OAM states that the signal propagate to become jM with j any integer. This smart technique to minimize losses caused by the pinhole has its own limitation and can only be leveraged to a certain extent. A larger step index M requires finer resolution and better error control in the fabrication of the SLMs. Such challenges may reduce the efficiency of the SLMs. Since the step in the OAM numbers is larger, the maximum OAM state that the cavity needs to support increases too. In Supplementary Figures 2a,b,c, the field intensity distributions of a few low OAM modes on the input/output mirror are shown for different M for the SLMs. It can be seen that a higher M together with an appropriate pinhole radius results in reduced photon loss.

Aside from its coupling to the input/output field, the $l = 0$ mode can also have additional leakage due to the coupling to $l \neq 0$ modes. The part of these modes that falls in the pinhole can escape quickly at a rate determined by the pinhole's coupling efficiency, which manifests as an additional loss

$$\gamma_0^{\mathrm{hole}} \approx 2(1-\eta^{\mathrm{hole}})\kappa \quad (23)$$

for the $l = 0$ mode. Here η^{hole} is a coefficient close to 1 determined by the portion of the higher modes' intensity distribution that falls outside the pinhole. Loss rate γ_j^{hole} for mode jM can be calculated similarly. It is expected that γ_j^{hole} decreases very quickly with j, since the leakage via the pinhole is much smaller for higher OAM modes.

Considering all major sources of loss discussed above, we have

$$\gamma_j = \gamma^{\mathcal{F}} + \gamma^{\mathrm{A}} + \delta_{0,j} 4\kappa + \gamma_j^{\mathrm{hole}} \quad (24)$$

for the j-th used OAM mode. Different terms in equation (24) are not equally important in determining the storage time of the quantum memory. As discussed earlier, $\gamma^{\mathcal{F}}$

is much smaller than other loss rates. The effect of the leakage via the pinhole is also limited. During the read-in phase, the low OAM modes are populated and they have some small leakage. However, such leakage does not have a dramatic effect on the storage time as long as the loss rate of these low OAM modes is much smaller than the group velocity of the signal in the OAM space. This is because, as shown in Fig. 5 in the main text, before the main intensity of the signal pulse can be lost by leakage via the pinhole, it already travels to higher OAM modes whose leakage is much lower. In the storage phase, the signal is safely kept in high OAM modes where the leakage via the pinhole is very low.

With the state of current technologies, the remaining loss channels by the phase modulators and SLMs are then the limiting factors for the storage time of the quantum memory. Their effect is persisting, even during the storage phase when transitions between OAM modes by the two auxiliary cavities (see Fig. 5a in the main text) cancel each other and the signal is frozen in the OAM lattice (see Fig. 5e in the main text).

In calculating and plotting (see Fig. 5 in the main text) the evolution of the signal pulse in the OAM space by numerically solving equation (17), we assumed a relatively high loss rate $\gamma_0^{\rm hole} = 0.2\kappa$ for the $l = 0$ mode to get a conservative result. Since $\gamma_j^{\rm hole}$ decreases very quickly with j and the pinhole leakage is not the limiting factor for the storage time, the specific dependence of $\gamma_j^{\rm hole}$ on j has little effect on the results. We simply use an exponential decaying function $\gamma_j^{\rm hole} = \gamma_0^{\rm hole} e^{-|j|}$. In Supplementary Figure 2d, we plot the calculated storage efficiency $\varepsilon_{\rm f}$ for the quantum memory [8] as a function of the storage time τ under different values of $\eta^{\rm A}$ and $\eta^{\rm hole}$. It is seen that the overall efficiency of the phase modulators and SLMs, $\eta^{\rm A}$, has a substantial impact on the storage time. Appreciable improvement can be achieved by lowing the loss of the phase modulators and SLMs.

Supplementary Note 4: Estimation of the filtering shape factor

The filter function for the optical filter is defined as the ratio between the output power and input power. Assuming moderate efficiencies for the phase modulators, SLMs, and pinhole, $\eta^{\rm A} = 95\%$ and $\eta^{\rm hole} = 95\%$, we have $\gamma^{\rm A} \simeq 0.1\kappa$ and $\gamma_j^{\rm hole} \simeq 0.1\kappa e^{-|j|}$. With a typical value of 10MHz for κ, we calculate the filter function in the presence of imperfections discussed in Supplementary Note 3, and plot the results in Fig. 6 in the main text. The calculation shows that, for a filter with a stop-band width of $4\kappa \simeq 40$MHz, a high skirt slope of 20dB/2MHz can be obtained, leading to a shape factor of 0.85.

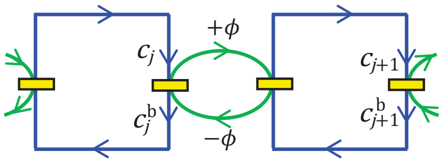

Supplementary Figure 1: **Effective 1D array of coupled cavities**. The field amplitudes c_j and $c_j^{\rm b}$ are defined at one beam splitter.

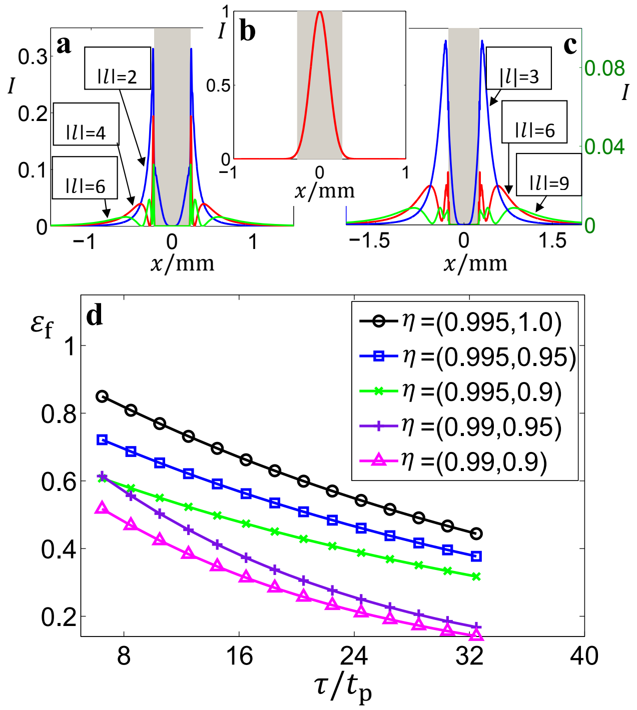

Supplementary Figure 2: **Transverse field distribution and storage efficiency for different values of** M. The cavity mirrors' focal length, the wavelength of the optical signal, and the width of the signal's beam waist, are 7.5cm, 880nm, and 0.12mm, respectively. The beam splitters and SLMs are assumed to be placed where the beam waist is located. (**a**) Transverse field distribution along the x-axis on the input/output mirror plane x-y, calculated using Collins integral [15] with a step index $M = 2$ for the SLMs. I is field intensity. The gray area marks the low-reflectivity pinhole whose radius is 0.225mm. The resulting efficiency is $\eta^{\rm hole} \simeq 90\%$. (**b**) Transverse field distribution of the $l = 0$ mode, most of which falls within the pinhole. (**c**) The same as in **a**, except that the step index of the SLM is $M = 3$, and the radius of the pinhole is 0.26mm. The efficiency is about 95%. (**d**) Calculated storage efficiency $\varepsilon_{\rm f}$ versus storage time τ. The input pulse is $E_{\rm in}^0 = \exp(-\frac{t^2}{2t_{\rm p}^2} - i\omega_0 t)$, with $t_{\rm p} = 2.5\kappa^{-1}$ and $\eta = (\eta^{\rm A}, \eta^{\rm hole})$.

Supplementary References

[1] Poon, J. K. S., Scheuer, J., Xu, Y. and Yariv, A. Designing coupled-resonator optical waveguide delay lines. *J. Opt. Soc. Am. B*, **21,** 1665–1673 (2004).

[2] Bayindir, M., Temelkuran, B. and Ozbay E. Tight-binding description of the coupled defect modes in three-dimensional photonic crystals. *Phys. Rev. Lett.*, **84,** 2140 (2000).

[3] Hartmann, M., Brandao, F. and Plenio, M. Strongly interacting polaritons in coupled arrays of cavities. *Nature Phys.*, **2,** 849–855 (2006).

[4] Luo, X.-W., *et al.* Quantum simulation of 2d topological physics in a 1d array of optical cavities. *Nature Commun.* **6,** 7704 (2015).

[5] Yariv, A. and Yeh, P. *Photonics: Optical Electronics in Modern Communications* (Oxford University Press, Oxford, 2007).

[6] Hernández, G. *Fabry-perot interferometers* (Cambridge University Press, Cambridge, 1986).

[7] Walls, D. F. and Milburn, G. J. *Quantum optics* (Springer-Verlag, Berlin, 2008).

[8] Simon, C., *et al.* Quantum memories. *The Eur. Phys. J. D* **58,** 1–22 (2010).

[9] Yanik, M. F. and Fan, S. Stopping light all optically. *Phys. Rev. Lett.* **92,** 083901 (2004).

[10] Nagorny, B., Elsässer, T. and Hemmerich, A. Collective atomic motion in an optical lattice formed inside a high finesse cavity. *Phys. Rev. Lett.* **91,** 153003 (2003).

[11] Leidinger, M., Buse, K. and Breunig, I. Highly sensitive absorption measurements in lithium niobate using whispering gallery resonators. *Proc. SPIE* **9347,** 93471D (2015).

[12] Oemrawsingh, S. S. R., *et al.* Experimental demonstration of fractional orbital angular momentum entanglement of two photons. *Phys. Rev. Lett.* **95,** 240501 (2005).

[13] Marrucci, L., *et al.* Spin-to-orbital conversion of the angular momentum of light and its classical and quantum applications. *J. Opt.* **13,** 064001 (2011).

[14] Raut, H. K., Ganesh, V. A., Nair, A. S. and Ramakrishna, S. Anti-reflective coatings: A critical, in-depth review. *Energy Environ. Sci.* **4,** 3779–3804 (2011).

[15] Collins, S. A. Lens-system diffraction integral written in terms of matrix optics. *J. Opt. Soc. Am.* **60,** 1168–1177 (1970).
